# Three-dimensional Graphene with MoS_**2**_ Nanohybrid as Potential Energy Storage/Transfer Device

**DOI:** 10.1038/s41598-017-09266-2

**Published:** 2017-08-25

**Authors:** Kulvinder Singh, Sushil Kumar, Kushagra Agarwal, Khushboo Soni, Venkata Ramana Gedela, Kaushik Ghosh

**Affiliations:** Institute of Nano Science and Technology, Sec. 64, Mohali, Punjab India

## Abstract

Portable and matured energy storage devices are in high demand for future flexible electronics. Flowery shaped MoS_2_ nanostructures with porous and flake like morphology was used to study the supercapacitive nature with specific capacitance (C_*sp*_) of 169.37F/g, the energy density of 28.43 Wh/Kg and power density of 10.18 W/Kg. This nanoflower like architecture was decorated on 3D-graphene on Graphite electrode to design the solid-state-supercapacitor prototype device of dimensions of 23.6 × 22.4 × 0.6 mm^3^ having considerable high C*sp* of 58.0F/g and energy density of 24.59 Wh/Kg, and power density of 8.8 W/Kg. Four fabricated supercapacitors were connected in series for real state practical demonstration using the light emitting diode that remains enlightened for 40 s by charging it only for 25 s. This study demonstrates the 3D-graphene/MoS_2_ nanohybrid has a quite high overall potential window nearly about 2.7 V (−1.5 to +1.2 V) in KOH-PVA medium which can be used for the development of solid-state supercapacitors thereby completely eliminating the need for any expensive ionic liquid mediums thus building an exciting potential for high-performance energy storage/transfer devices.

## Introduction

Current economy and population growth rates project a demand for energy of 40 terawatts (TW) with a total population of 10 billion by 2050^1^. Due to the increase in demand for energy, the non-renewable sources like fossil fuels are continuously decreasing and will automatically come to an end in the near future. To meet this demand, alternate renewable energy sources has been projected like solar, wind and hydropower. However, a suitable storage device like Li-ion batteries and Na-air batteries and so on are currently lagging behind with a storage capacity of only 1% of the total renewable energy with longer charging time. Presently, batteries are used in wide variety not only for energy storage but also for the energy supply devices^2^. The use of a battery in large scale sophisticated electronic devices is limited due to its heavy weight, portability, flexibility and other issues^3, 4^. Currently, researchers are looking for alternative energy storage devices that effectively store and provide energy as per requirement. Supercapacitors are portable, flexible^5^ and transparent unlike conventional batteries that draw the attention of the researchers to extract its interesting properties. Along with these physical advantages, supercapacitor has high charge storage capacity, high power density, low energy density, longlife time and short charging time as compared to traditional batteries^6–8^. A notable improvement in performance has been achieved through recent advances in understanding charge storage mechanisms and the development of advanced nanostructured materials in supercapacitor devices^6, 9^. Supercapacitors are classified mainly in two categories such as, pseudocapacitor and electrical double layer capacitance (EDLC)^8^. In case of pseudo capacitor, the electrical energy is stored via faradaic reaction in electrolyte and electro-active species on the electrode surface. There are several reports in the literature based on pseudocapacitors made of various nanomaterials as well as their composite, such as SnO_2_-V_2_O_5_, Cobalt pyrophosphate, Metal oxide-CNT, RGO-metal sulfide, MnO_2_-veritcally aligned graphene, etc. having a specific capacitance in the range of 190–3480F/g with energy density ranging from 14–89 Wh/kg^10–12^. It has been reported that the capacitance due to faradaic peaks increases the specific capacitance of supercapacitor 10–100 times higher than the electrostatic capacitance of an EDLC. Recently, Ko *et al*.^13^. has demonstrated porous graphitic carbon with Ni_2_P_2_O_7_, heterostructure has shown high specific capacitance of 1893 F/g and Liao *et al*.^14^. has reported ultrahigh supercapacitance of 3480F/g using Co_3_O_4_ nanoparticles on vertically aligned graphene sheets supported in carbon fibers. Although pseudocapacitors are shown to have high initial capacitance, but due to limited durability, lack of stability during cycling and poor power density, its practical use in solid-state energy storage devices is limited^8, 15, 16^. In addition to this, crystallinity and morphology also play an important role in specific capacitance of pseudo capacitors, lower the crystallinity higher is the specific capacitance, which is due to the availability of chemically active dangling units which can easily take part in oxidation-reduction cycle^8^. To overcome these limitations, there is an extensive drive to develop EDLC type supercapacitor that carries various advantages over pseudo capacitor. In case of EDLC, electrical energy is stored over the layer of current collector without any redox couple. Due to the absence of faradaic current, EDLC type supercapacitors are more reliable and have shown long term durability with minimum loss of capacitance under multiple charging-discharging cycles.

Xu *et al*. have fabricated graphene hydrogel for the EDLC type supercapacitor where storage performance is related to the high surface area^17^. Sundaram *et al*. have fabricated MnO_2_ nanostructures for EDLC type supercapacitance in a lower applied potential window having C_*sp*_ of 50F/g^18^. Various carbonaceous materials till now have been developed for the testing of EDLC type supercapacitors due to its cost efficiency, high surface area, porous nature and versatile existing forms^19, 20^. Significant enhancement in capacitive performance is not only governed by the properties of electroactive materials but also by the separators and electrolytes. Lin *et al*. have fabricated graphene based supercapacitor (C_*sp*_ of 130F/g) in ionic liquid electrolyte^21^ where ionic liquids shows high chemical stability as compared to conventional electrolytes at a wide potential range^22^. Further development of the storage device lies on the various tricky parameters such as, proper choice of electrolyte, electrochemically stable current collector and thin porous spacer matrix between the electrode materials etc^3, 4, 23, 24^. The current developmental trend of miniaturized autonomous electronic equipment such as implantable medical devices and active radio frequency identification (RFID) tags have raised the demand for solid-state-supercapacitors^25^. Significant efforts have been devoted to improve flexibility and energy storage/transfer capacity of solid-state supercapacitors based on various carbonaceous materials and their composites^26^, out of which graphene shows a promising choice of material due to high conductivity, flexibility, transparency, large surface area and layer like structure that drags attention to various scientific communities for its large scale application in energy storage devices^17, 23, 24^. In most of the cases, graphene based solid-state-supercapacitors demonstrate very low specific capacitance which is mainly due to the parallel restacking of graphene sheets resulting in decrease of the specific capacitance^17, 27, 28^. Recently, 3D graphene proves to be more prominent material for solid state supercapacitor due to the partial stacking of graphene sheets in random orientation in three dimensions resulting in formation of micro porous structures^17^. This unique orientation of graphene not only helps to improve the charge storage capacity of graphene but helps the electrolytic ions to move freely via porous geometry. To further improve the response of the 3D graphene as solid-state-supercapacitor various layer/porous nanomaterial hybrids have been reported which develops the charge storage capacity of the 3D graphene via increase in surface area and porous nature of the hybrid materials^29–33^. It is already reported that graphene based hybrid material possess superior charge storage capacity by providing enhanced surface area, electrical conductivity, thermal stability and mechanical strength to graphene^34^. Out of the various nanomaterials, 2D transition-metal dichalcogenides have been a perfect candidate for charge storage applications due to their layer like structures that shows weak Van der Waal force of attraction with graphite or graphene, especially 1T-MoS_2_ nanostructures^37–41^. Due to their band gap of ~1.9 eV, which reveals the semi-insulating nature, they are thus not immediately thought to be an electrode material for energy storage application^42^. But still MoS_2_ nanosheets display an excellent performance in wide, negative potential in neutral electrolytes, such as high specific capacitance and rate performance, which promotes the MoS_2_ nanosheets to be a promising electrode material for supercapacitor applications^43^. Till date various MoS_2_ based supercapacitor have been developed but only handful reports have been presented which shows the practical applicability of solid state supercapacitor^42–46^. The main research challenges associated with electrochemical supercapacitors are to develop new electrode materials, to enhance specific capacitance by modification of the electro- active material^9, 37^.

In this work, we report the simpler and cost effective process via chemical route of synthesis of hybrid MoS_2_ nanoflowers with 3D graphene heterostructure as an active material for energy storage device. The real demonstration of the solid state supercapacitor has been performed here using the 3D graphene-MoS_2_ hybrid over graphite current collector. Herein, the specific capacitance of bare MoS_2_ nanostructures comes out to be 169.37F/g with an energy density of 28.43 Wh/kg and power density of 10.17 W/kg. The capacitive performance of 3D graphene-graphite electrode has been improved significantly when the current collector is incorporated with MoS_2_ nanoflowers. This remarkable supercapacitor performance of the hybrid could be attributed to the combined effect between layered MoS2 and 3D graphene which could have some real phase application in modern energy storage devices.

## Results and Discussion

### Characterization of 3D graphene and MoS_2_ nanoflowers

**Figure 1:**
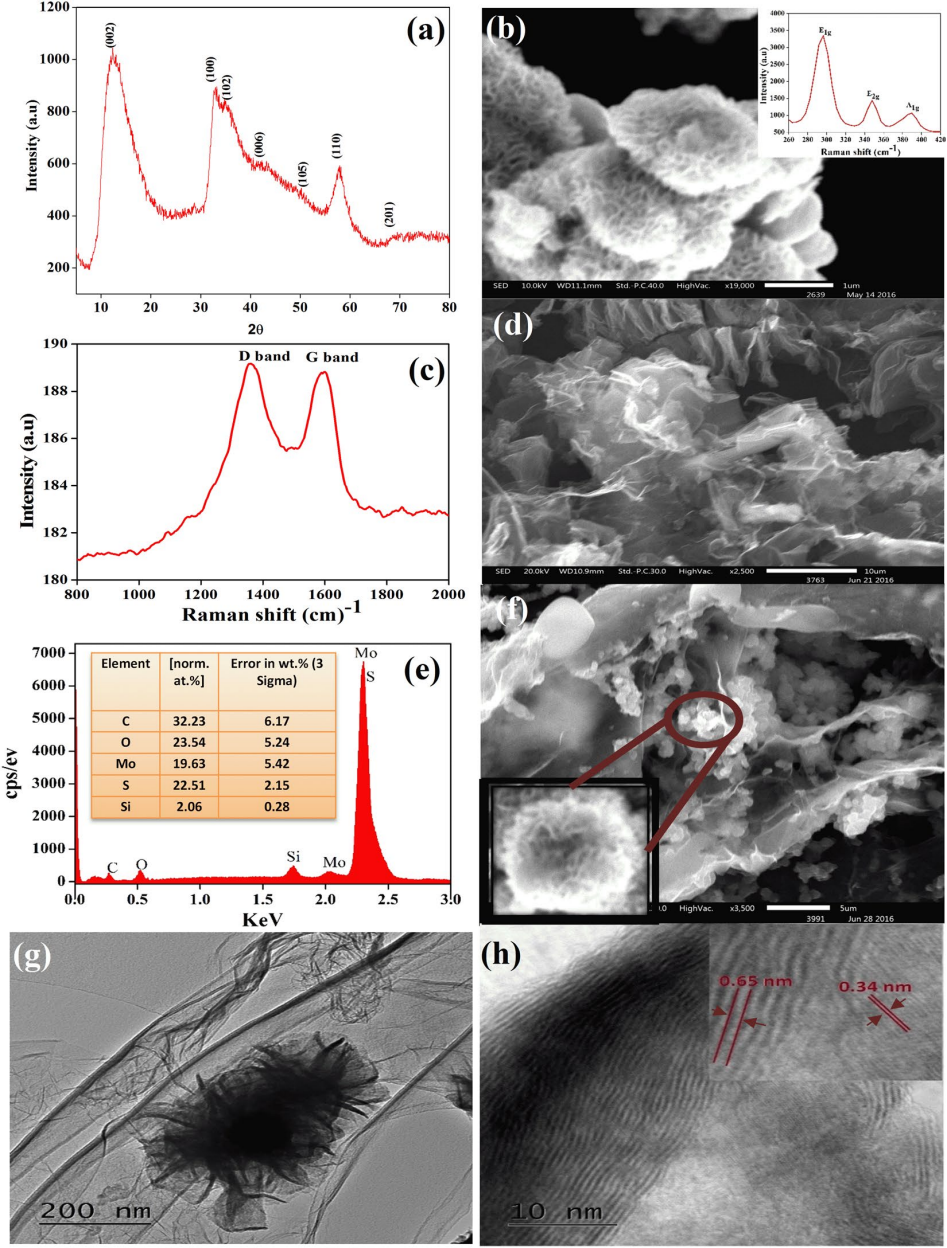
(**a**) Diffraction pattern of as-prepared MoS_2_ nanoflowers with peaks at 13.55, 33.06, 35.22, 42.5, 49.82, 58.01, and 69.77. (**b**) SEM image of MoS_2_ nanoflowers (Inset Raman Spectrum of MoS_2_ nanomaterial with the band at 296.86, 346.87 and 390 cm^−1^). (**c**) Raman band at 1357 and 1596 cm^−1^ of 3D graphene. (**d**) SEM images of 3D graphene. (**e**) EDX spectrum of MoS_2_-3D graphene hybrid. The flower like MoS_2_@3D-graphene hybrid architecture has also been observed in SEM and high resolution TEM images shown in (**f**) and (**g**), respectively, which reveals the petals of MoS_2_ nano-flowers along with the 3D-graphene network effectively enhance the overall surface area that impacts on high storage capacity, (**h**) The hybrid interface is further confirmed by the HRTEM analysis where the lattice spacing are calculated to be 0.65 nm for MoS_2_ and 0.34 nm for graphene, respectively.

As-prepared MoS_2_ nanoflowers were characterized by XRD, SEM, and Raman spectroscopy. Figure 1(a) displays the XRD pattern of the MoS_2_ nanoflowers. It shows the diffraction patterns at 2θ = 13.55, 33.06,35.22, 42.5, 49.82, 58.01, and 69.77 which arises from the (0 0 2, (1 0 0), (1 0 2), (006), (105), (110) and (201) planes of MoS_2_^47^. The observed diffraction patterns are broadened in nature with a slightly lower diffraction angle shift as compared to the bulk 2H-MoS_2_, suggesting the presence of randomly stacked layers of MoS_2_ which may be due to the intercalation of NH_3_ in between the layers during the synthesis^48^. The XRD pattern well matches with the already common works on randomly stacked MoS_2_ nanostructures^49, 50^. No peak other than MoS_2_ has been observed in the XRD pattern revealing that the synthesized MoS_2_ nanoflowers are highly pure in nature. Figure 1(b) shows the SEM image of MoS_2_ nanoflowers at a low magnification, it is observed that the particles are spherical in nature with porous nanostructure in the order of nanometers. It is well established in the literature that porous nanomaterials possess a superior charge storage capacity as compared to that of their bulk counterpart^51^. The formation of the porous MoS_2_ nanoflowers is due to the reaction conditions as well as the precursors used in the reactions. Coagulation into a sphere is most likely due to the hydrothermal reaction conditions and the precursors used. During the reaction, ammonium molybdate releases MoO^4−^. These ions, having a layered structure, react with sulfide ions and intercalation by the residual ammonia prevents the stacking of MoS_2_ layers which leads to the self-assembly of a sphere-like morphology^48^. The Raman spectrum of MoS_2_ nanostructure was carried out at room temperature (Inset Fig. 1(b)). Three distinct characteristic band positions were observed in the raman spectrum of MoS_2_ nanostructures i.e. E_1g_, E_2g_ and A_1g_ at 296.86, 346.87 and 390 cm^−1^ respectively. The raman band appearing at 296.86 cm^−1^ with strong intensity (E_1g_ symmetry), arises due to S atom in the basal plane. Band appearing at 347.86 cm^−1^ (E_2g_ symmetry) arises due to intralayer vibrational mode of Mo and S atoms in the basal plane. The A_1g_ mode which appears at 390 cm^−1^ is due to the intralayer mode involving the motion of S atoms^52, 53^. Figure 1(c) depicts the Raman spectrum of 3D graphene in which two prominent raman bands were clearly visible that corresponds to D and G band at 1357 and 1596 cm^−1^, respectively^54–56^. The appearance of D band arises due to the activation of the first order scattering process of sp^3^ hybrid carbons, which is attributed to dislocation defects in graphene sheets^57^. Similarly, the SEM studies of as-synthesized 3D graphene were carried out, see (Fig. 1(d)). Graphene hydrogels are formed by the partial overlapping of graphene sheets via hydrophobic and π-π interaction in the 3D space helps it to possess an interconnected porous network. The elemental analysis of 3D graphene-MoS_2_ hybrid was carried out by EDX studies (Fig. 1(e)). Elemental analysis shows that the hybrid contains C, Mo and S elements. In addition, Si element was also detected in the EDX studies which appear due to the usage of Si wafer for the elemental detection. Inset of Fig. 1(e) reveals the complete percentage composition of the hybrid materials, where the wt% of C, O, Mo, S, and Si are 32.32, 23.54, 19.63, 22.51 and 2.06 respectively. For morphological characterization of 3D graphene-MoS_2_ hybrid, SEM studies were carried out.

Figure 1(f) shows that the MoS_2_ nanospheres, as well as 3D graphene, were successfully deposited over graphite. Inset of Fig. 1(f) shows the flaky nature of MoS_2_. SEM images clearly indicate that the MoS_2_ has completely formed a hybrid network with 3D graphene. Figure 1(g,f) displays the TEM images of 3D graphene-MoS_2_ hybrid nanomaterial revealing the flower like morphology of MoS_2_ nanostructure decorated on 3D graphene hybrid and is fully consistent with SEM results.

The MoS_2_ nanosheets along with 3D-Graphene will noticeably improve the number of the exposed electrochemically active sites, which will considerably enhance the ion diffusion efficacy during the reversible electrochemical reactions^58^. There is a possibility to enhance the energy density of supercapacitor by tuning the porosity of the active material which should be lower than the hydrodynamic size of the active ion or equivalent or higher than the desolvated ions^6^. This porous nature of the active material will help to reduce the rate of discharging hereby enhancing the energy density of supercapacitor. Further deep insight of the nanomaterial (Fig. 1(h)) demonstrate the 3D graphene-MoS_2_ interface and the characteristic lattice fringes, shown in HRTEM image, corresponds to 0.65 nm of MoS_2_ and 0.34 nm of graphene respectively, that are attributed to the (002) planes of MoS_2_ and multilayer graphene^59^.

### Cyclic Voltammetry studies of MoS_2_, 3D graphene and hybrid in solution phase

**Figure 2:**
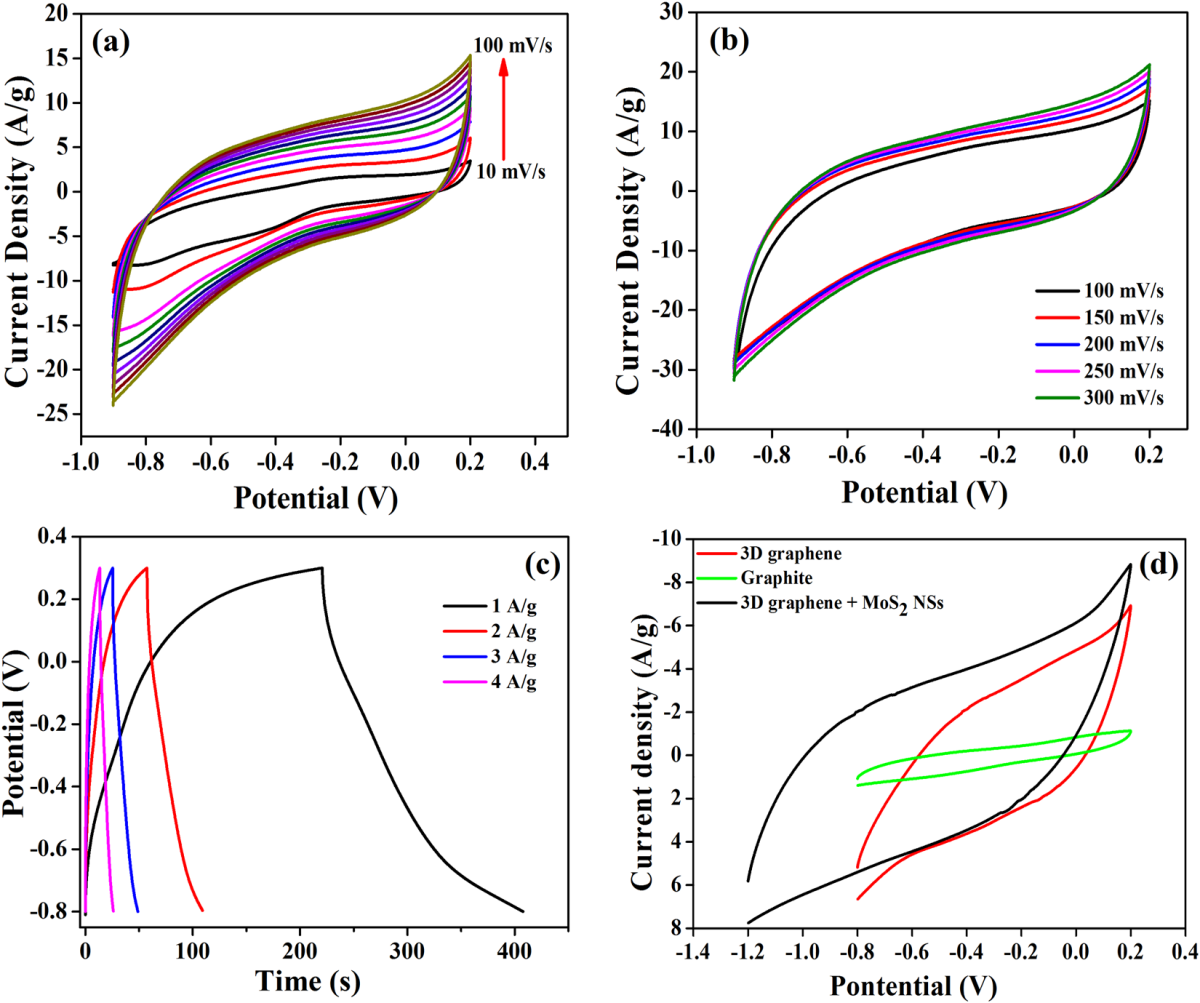
(**a**) Cyclic voltammogram of MoS_2_ modified electrode in the presence of 0.1 M KOH solution in low scan rate (10–100 mV/s), (**b**) Cyclic Voltammograms of MoS_2_ modified electrode at higher scan rates (100–300 mV/s). (**c**) Galvanostatic charge/discharge curves of MoS_2_ modified electrode in 0.1 M KOH solution. (**d**) Comparison of 3D graphene, MoS_2_ and 3D graphene-MoS_2_ hybrid response over 1 × 1 cm^2^ graphite piece in 0.1 M KOH.

Figure 2(a) displays the cyclic voltammetry response of MoS_2_ modified glassy carbon electrode in 0.1 M KOH solution from low (10 mV/s) to higher (300 mV/S) scan rate. From the CV it is clear that no significant redox peak was observed which reveals that MoS_2_ nanostructure behaves as typical electrical double layer capacitance which is similar to already published in the literature^41, 48, 60^. Also the working potential of MoS_2_ nanoflowers were quite high i.e. −0.9 to 0.2 i.e. 1.1 V of working potential was observed. The specific capacitance of MoS_2_ was calculated using given equation:

1 $$\frac{{\int }^{}IdV}{\upsilon \times {\rm{\Delta }}V\times m}$$

where I is the current, ʋ the scan rate ΔV is the working potential window and m is the active mass of the material present on the surface of the electrode^61^. By this equation, the specific capacitance of MoS_2_ nanostructure comes out to be 145.73F/g (100 mV/s).

The energy density of the present system was calculated by using equation^62^:

2 $$E=C{\rm{\Delta }}{V}^{2}/2$$

where E is the energy density, C is specific capacitance, ΔV is the working potential. To calculate the power density equation 3 was used ref. 19:

3 $$P=E/t$$

where P is the power density and t is the discharge time.

Figure 2(b) displays the cyclic voltammetry response of MoS_2_ nanospheres in 0.1 M KOH solution at various scan rates. As seen from the figure the approximately rectangular shape with high symmetry is an indicator to double layer capacitance with fast charge-discharge rates. These results i.e. electrical double layer formation, are in good agreement with the previously published literature^47, 48, 63^. For various scan rates i.e. 100, 150, 200, 250, 300 mV/s, the specific capacitance using equation (1) comes out to be 145.73, 124.31, 116.58, 110.39, 104.79 F/g respectively. With the increase in scan rate, the current density increases revealing the existence of ideal supercapacitor behaviour and specific capacitance decreases thus indicating that at lower scan rates the electrolyte ions diffuse into the inner layers of the active material quite easily, leading to higher interactions with the active sites of the active material for the charge transfer (Fig. S1 in Supplementary)^64^. Effect of scan rate on the energy density was displayed in Fig. S2 of Supplementary. These results indicate that MoS_2_ is a very important candidate for supercapacitor applications. Further for specific capacitance calculation charge discharge was carried out in between the potentials of −0.8 to 0.2 V (Fig. 2(c)). From charge discharge studies, it is observed that the charging time for MoS_2_ nanoflowers is ~200 s and for discharging it is ~190 s which reveals the MoS_2_ nanoflowers show good charging and discharging behavior. For further calculation of specific capacitance by charge-discharge scan Fig. 2(c), following relation was used:

4 $${C}_{sp}=I{\rm{\Delta }}t/m{\rm{\Delta }}V$$

where I is the discharge current (mA), Δt is the discharge time (s), m is the mass of the electro-active material (mg) and ΔV is the potential window.

The specific capacitance obtained by from CV curves is about 169.37, 95.66, 62.52 and 46.17F/g for 1, 2, 3 and 4 A/g respectively. This observed specific capacitance shows a significant improvement over previously reported values^65^ and is in good agreement with the specific capacitance using equation (1) ^40, 48^. For comparison, the supercapacitor performance of 3D graphene, graphite and MoS_2_ have been explored over 1 × 1 cm^2^ graphite sheet (Fig. 2(d)). Green, red and black line denotes the response of graphite, 3D graphene and hybrid material (3D graphene and MoS_2_) in 0.1 M KOH. By comparing their respective cyclic voltammograms, it is observed that the hybrid material not only increases the working potential window but also increases the current response, C_*sp*_ and energy density for graphite, 3D graphene over graphite and of 3D graphene-MoS_2_ hybrid comes out to be 7.83, 46.82, 58.0 F/g and 1.09, 13.0, 24.59 Wh/Kg, respectively which shows that the hyrid material has more supercapacitive performance as compared to both 3D graphene and graphite material. The complete values of specific capacitance, energy density, and power density was given in Tables 1 and 2 of Supplementary Information.

**Table 1:** Comparative studies of recently reported solid state supercapacitor utilizing various electroactive materials.

Nanomaterial	Energy density	Power density	Capacitance (F/g)	Reference
Co_3_O_4_	80 Wh/Kg	20 W/Kg	3560	14
RuO_2_.nH_2_O	10.62 WhJ/g	4.456 kW/Kg	570	71
BCN			244	72
Carbon with PHEMA-co-TMPA	—	—	130	73
CNT and PEDOT	8.85 mWh/cm^3^	9.4 W/cm^3^	0.354	74
Carbon cloth with phosphoric acid doped polybenzimidazole	10 Wh/kg	300 W/kg	290	75
High specific surface Area (SSA) carbon and poly [2,5-benzimidazole]	—	—	248	76
polyaniline and sulfonated polymers	—	—	98	77
Graphene	82 kW/kg	32.3 W/Kg	98	78
rGO	34 µWh/cm^2^		56.11	79
3D graphene-MoS_2_ hybrid	24.59 Wh/Kg	8.8 W/kg	58 F/g	Present work

### Solid state supercapacitor studies

**Figure 3:**
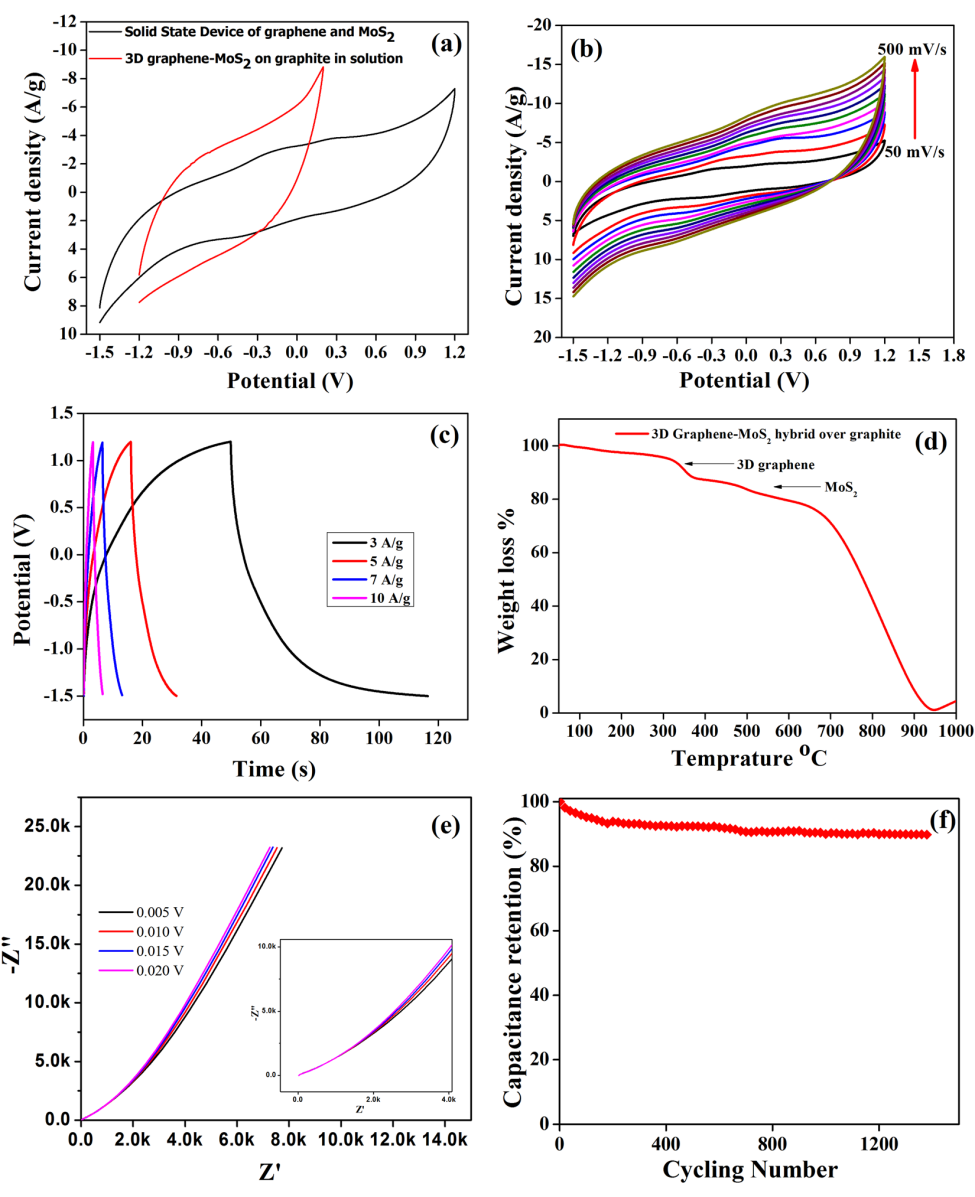
Schematic process flow for the fabrication of device using graphite as current collector. On top of graphite 3D graphene was grown further which was kept in 5 mg/mL solution of MoS_2_ of isopropyl alcohol and finally by using PVA-KOH solution two plates of graphite were assembled to prepare real solid state supercapacitor.

Figure 3 displays the complete schematic of fabrication of solid state device. For synthesis of solid state device first the graphite sheet of dimension 2.2 × 2.2 cm^2^ was scotch taped and then put in teflon line stainless steel vessel, 5 mg/mL solution of graphene oxide was added along with 2 M ascorbic acid solution and afterwards hydrothermal was sealed. The hyrothermal bomb was then kept at 120 °C for 4 h resulting in the formation of hydrogel of 3D graphene over graphite piece. This modified graphite was kept in 5 mg/mL solution of MoS_2_. Similiary other plate of supercapacitor was fabricated using same protocol. After this the two plate was assembled by using filter paper soaked with PVA/KOH solution.

**Figure 4:**
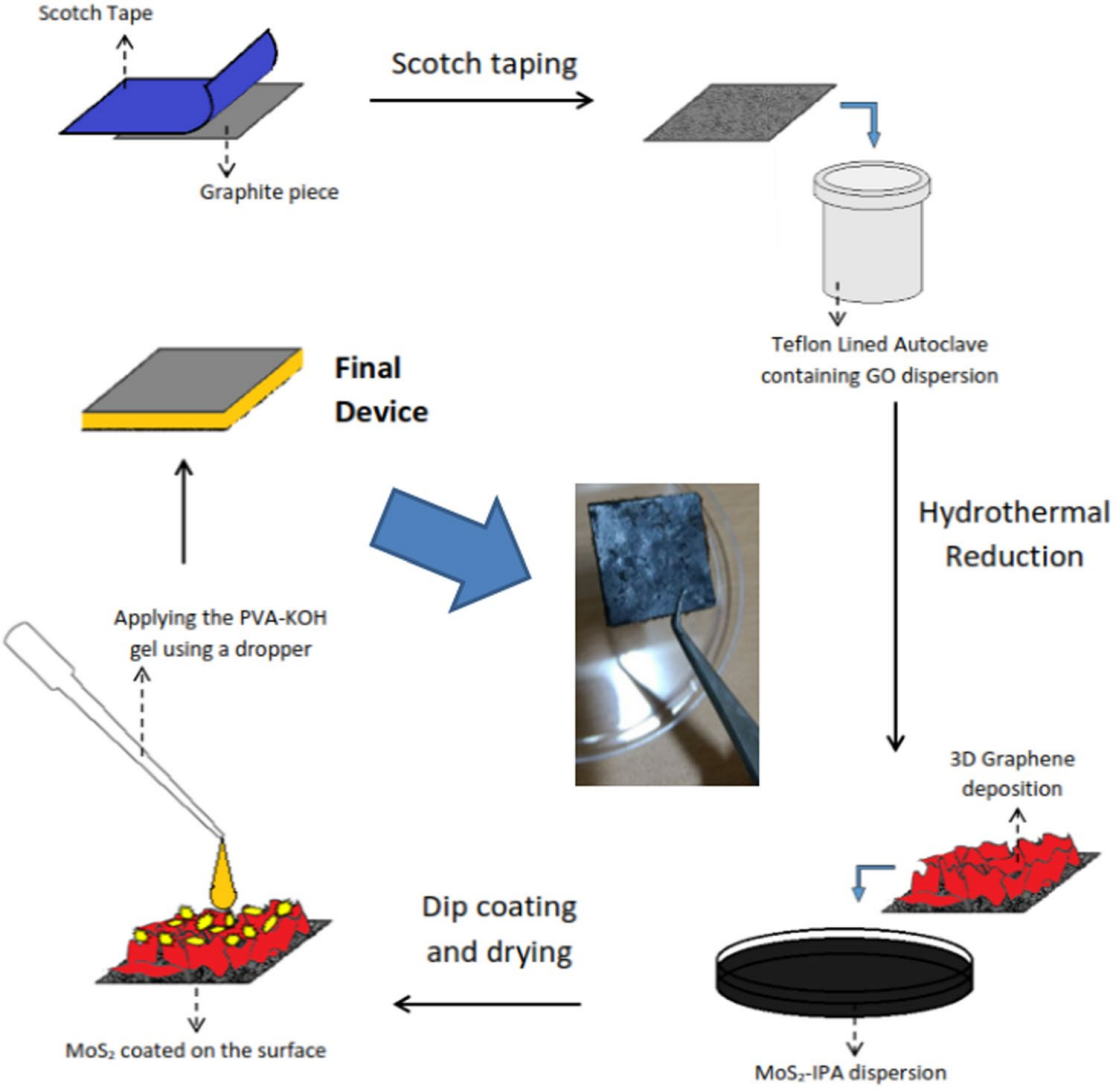
Comparison of MoS_2_-3D graphene hybrid in solution and solid state supercapacitor (**b**) scan rate studies of device (**c**) charge discharge studies. (**d**) TGA analysis of the hybrid material over graphite. (**e**) Nyquist plot for MoS_2_-3D graphene hybrid, (**f**) Cycle performance measured at 100 mV/s, and measured loss of stability is found to be 10% after 1400 cycles.

Successful implementation of the supercapacitor in solid-state without a loss in performance can be inferred. Figure 4(a) depicts comparative cyclic voltammetry curves of the fabricated solid-state supercapacitor as well as the hybrid electroactive material in solution. This reveals that area under the curve almost doubles due to the contribution of two parallel electrode plates in the device with large potential window ranging from -1.5 V to 1.2 V. It can be clearly understood, that the solid-state supercapacitor is performing quite well as compared to the hybrid material in solution. The potential window as well as the area under the curve has increased significantly in solid-state devices, due to the combination of two electrode parallel plates, thus indicating that the hybrid materials shows potential development of supercapacitive performance over 3D-graphene and graphite based electroactive materials. Specific capacitance for 3D graphene-MoS_2_ comes out to be 74.94F/g and the specific capacitance of 3D graphene-MoS_2_ hybrid in solid state device comes out to be 58.0F/g (using equation 1). Similarly using equation (2) energy density comes out to be 15.77 and 24.59 Wh/kg for solution and solid state device, respectively. The performance of the hybrid material over graphite plate at various scan rates was also studied, shown in Fig. 4(b). Similar to MoS_2_, no oxidation reduction peak appeared in the device indicating the existence of pure EDLC type behavior. The current density of the hybrid material increases with the increase in scan rate, confirming its ideal nature. A large operating current indicates that the existence of low internal resistance between 3D graphene to graphite electrode. This is due to the excellent electrical conductivity of graphene sheets and the absence of electrical barrier at the 3D graphene-graphite sheet interface via π-π interactions. For the solid-state supercapacitor, two sheets of 2.2 × 2.2 cm^2^ graphite, coated with 3D graphene-MoS_2_ hybrid were pasted using a filter paper soaked in PVA-KOH gel and kept for drying in a vacuum desiccator. These results indicate that the performance of the solid-state supercapacitor is quite high as compared to that in solution and that it can work as a standalone solid-state device. It is observed from the galvanostatic charge-discharge curves in Fig. 4(c) that the charging and discharging time of the solid-state device is found to be significantly shorter compared to that of MoS_2_ nanoflowers (see Fig. 2c). This is suggesting a marked improvement in supercapacitor performance due to the presence of 3D graphene. In Fig. 4(c), it is also noted that the charging time (~50 s) is lower than the discharging times (~68 s), emphasizing upon the ideal supercapacitor behavior of the device. Further, at high current density the galvanostatic charge discharge (GCD) curves show typically triangular shape which is the characteristic of nearly ideal capacitive performance. However, at low (<3 A/g) current density the deviation of the GCD pattern is attributed possible due to the redox reaction occurs at the dangling bond of the 3D graphene and defect site of the MoS_2_, which is quite similar to the recent report^9, 66, 67^. The formation of the plateau at low current density at high applied voltage (2.7 V) fully opens the stacked 3 D graphene as well as MoS2 nano flaks which allow ion intercalation and creates abundant ion-accessible sites for adsorption/intercalation, and dramatically increases the cell’s capacitance^68^.

The weight calculation of the active material was done by TGA analysis. Figure 4(d) displays the TGA study of MoS_2_ 3D graphene hybrid over graphite. TGA spectrum shows two weight losses one at ~350 °C and other at around 500 °C. Weight loss i.e. 5% at 350 °C corresponds to 3D graphene^69^. While the weight loss i.e. 2.5% at around 500 °C corresponds to MoS_2_ nanoflowers^59^. From the TGA calculation, the weight of the active material comes out to be 1.1 mg, which has been used to calculate the specific capacitance (using eq. 1) of 58.0F/g.

To further understand the superior performance of the MoS_2_-3D graphene hybrid electrode, electrochemical impedance spectroscopy (EIS) is performed by electroactive materials to analyze the kinetic feature of the ion transportation and ion diffusion in the porous hybrid electrode in aqueous electrolyte system. Figure 4(e) shows the Nyquist plot obtained at the frequency range from 100 kHz to 0.1 Hz with different amplitude of single sin wave (0.005, 0.01, 0.015, and 0.02 V RMS value). The calculated internal resistance (R_s_) for MoS_2_-3D graphene hybrid is 18.66 ohm. The corresponding equivalent circuit has been shown in Figure S5 in Supplementary Information. A sharp increase of the impedance plot at low frequencies indicates the capacitive behavior of the electrode. It is observed that like an ideal double-layer capacitor, the plot comes out to be a vertical line, parallel to the imaginary axis^70^. The enchantment in energy density is found to be attributed to the enlarge operating potential voltage of 2.7 V in the gel electrolyte (PVA with KOH) and the relatively high specific capacitance of both electrodes, especially the negative electrode, was found which is very important for the solid state devices. The stability curve strongly indicates that about 90% of the initial capacitance can still be retained after 1400 cycle at the scan rate of 100 mV/s (Fig. 4f), refers to the superior cycling performance.

**Figure 5:**
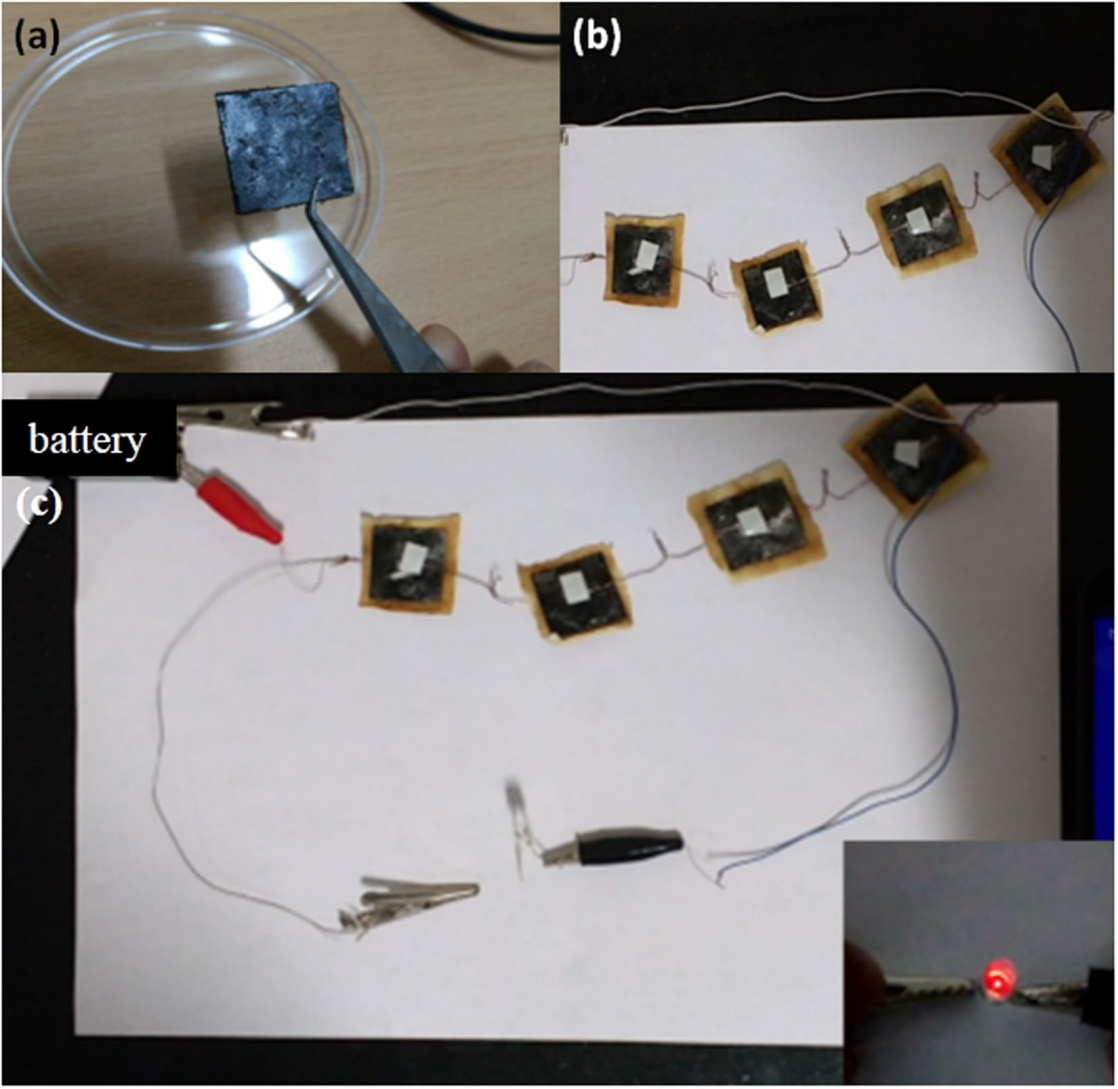
(**a**) Digital image of the single device. (**b**) Four devices fabricated and connected in series. (**c**) Digital image of the four devices connected in series (Charging for 25 s), Inset figure digital image of LED glow (Discharging for 40 s).

Figure 5(a) displays the digital image of fabricated solid state device. Finally, the solid state supercapacitor response has been monitored by using red LED. Four devices were connected in series to monitor the response of red LED. The devices were charged with 9 V battery for 25 s.

After that the LED was connected to the devices, the response was monitored through LED glow. With 25 s of charging the LED glows for 40 s. The video recording is added to the Supplementary data. This reveals that the present fabrication of the device completely work as solid state supercapacitor. Figure 5(b) displays the charging of four devices in series connection and the inset represents the response of the LED after charging of the solid state supercapacitor. The practical applicability of the device is demonstrated by the lighting of a red LED bulb. Four devices of the exact same dimensions and similar mass loading were connected in series and charged by a 9 V battery for 25 s. On connecting the device array to the LED, it remained lit up for an impressive 40 s before dying out. The longer discharge time compared to the charging time makes the device suitable for use in practical energy storage and transfer application.

The Table 1 depicts the comparative statements of recently reported solid state supercapacitors utilizing various electroactive materials along with our present findings. Although the specific capacitances of the devices made of active materials like RuO_2_, high-SSA Carbon and BCN, are quite high but the corresponding energy density is low that may be due to the shorter range of working potential. However, it is quite challenging to expand the working potential of the electrode for the practical use of device in real field of application. Common technique is to utilize the ionic liquid based electrolyte like BMIM, which screens the ionic interaction of ions with C ≡ N moieties and make them free to move. This may assist the fast ion transport. Thus, expanding the working potential without introducing ionic liquid can lead to a successful attempt for the development of solid state supercapacitor. The carbon cloth with phosphoric acid doped polybenzimidazole, Co_3_O_4_ and polyaniline with sulfonated polymers show significant enhancement of C_*sp*_ due to pseudocapacitance, which is better to avoid for long term durability of the practical devices. The present report focus on the 3D graphene-MoS_2_ hybrid for the development of real solid state supercapacitor which not only works well in negative potential but also expands significantly in positive working potential. The overall potential window for solid state supercapacitor is quite high which is about to be 2.7 V (i.e. −1.5 V to +1.2 V) in KOH-PVA medium without utilizing any expensive ionic liquid mediums. Recently a solid state architecture has been designed using a similar heterostructure^66^, however a costlier growth technique has been adopted where for the graphene growth was performed in 1000 °C in CVD setup. In contrast our study shows a very simpler and cost effective process via chemical route to design heterostructure. In comparison to the device performance, they report the specific capacitance of 19.44 F/cm^3^ in contrast to our device as 58.0 F/g. Here, it is noticed that the straight forward comparison is not feasible because the analytical approaches are entirely different.

## Conclusions

MoS_2_ was successfully paired with 3D graphene to fabricate a flexible, solid-state supercapacitor with outstanding performance. The facile method of fabrication can easily be scaled up at minimal cost to create a large array of supercapacitors which is lightweight and reliable. An immediate application can be found in solar energy storage due to its large energy storage capacity in a short span of time in purely EDLC type nature. The specific capacitance of the solid state supercapacitor comes out to be 58 F/g with energy density of 24.59 Wh/Kg and power density of 8.8 W/kg. It can be a partial replacement of conventional batteries and can be easily incorporated into flexible and thin electronics due to its high stability, thinness and flexibility. The operating potential window can be further enhanced by using an ionic-liquid gel-based electrolyte. Other transition metal dichalcogenides can also be investigated along with MoS_2_ for their supercapacitor performance on top of 3D graphene matrix.

## Experimental Section

### Materials

Graphite, ammonium molybdate, ascorbic acid, isopropyl alcohol, thiourea were purchased from sigma aldrich. All the solutions were prepared in deionized water. KOH was purchased from TCI chemicals.

### Synthetic and fabrication procedures

#### Synthesis and Purification of MoS_2_

The MoS_2_ nanoflowers were synthesized by a hydrothermal method using ammonium molybdate and thiourea as a starting precursor. Briefly, 1 M solution of ammonium molybdate and 5 M solution of thiourea were prepared separately and transferred into a teflon-lined stainless steel autoclave (100 mL capacity) and hydrothermal reaction was carried out at 180 °C for 24 h. Black precipitate was obtained followed by washing with deionized water and absolute ethanol. The washing procedure is repeated several times until the final product is free of any trace amount of residual ions. The final product was dried at 80 °C for 12 h.

#### Modification of the electrode

The as-synthesized MoS_2_ nanospheres were coated on a polished GC electrode surface (GC, diameter = 2 mm) to modify the Glassy Carbon (GC) electrode. For the coating, a solution of 1 mg/mL of MoS_2_ was prepared in isopropyl alcohol from which 5 µL was drop casted over the GC electrode surface. The prepared electrode was kept in a vacuum desiccator for 24 h to form a uniform layer over the surface. Cyclic voltammetry was carried out using a CHI electrochemical workstation with a three electrode system. While the MoS_2_-modified electrode was the working electrode, Ag/AgCl in sat. KCl was used as the reference electrode and platinum electrode was the counter electrode. These were dipped in 0.1 M KOH electrolyte.

#### Preparation of square shaped graphite electrodes

We followed the modified hydrothermal reduction method for the deposition of 3D graphene onto the graphite electrodes. Briefly, two rectangular pieces were taken from a graphite sheet and they were dipped into 10 mL of 5 mg/mL aqueous dispersion of graphene oxide (GO). This was freeze dried at −20 °C for 12 h. After the solution temperature was brought to room temp, 0.6 ml of 2 M ascorbic acid (AC) was added to the graphene oxide aqueous dispersion and the mixture was sealed in a teflon-lined autoclave and heated in an oven at 120 °C for 4 h, followed by drying of the attached graphite pieces in an oven set at 60 °C for 6 h. Once the drying was completed, the graphite pieces were dipped into a 5 mg/mL dispersion of MoS_2_ in isopropyl alcohol for 12 h. The graphite pieces were finally taken out and dried in an oven at 60 °C for 12 h.

#### Fabrication of Solid-state Supercapacitor

0.5 g of poly-vinyl alcohol (PVA) was added in small amounts to 9 mL of boiling de-ionized water under constant stirring with a magnetic heater-stirrer. Once the PVA gel reached a uniform consistency and bubbles started to form, 1 ml of 0.5 mg/mL aqueous solution of KOH was added into the gel drop wise under constant stirring. On attaining homogeneity, a piece of whatman filter paper was cut, soaked in the gel and placed in between the two electrode pieces. This arrangement was left to dry for 12 h and hence, the final device was completely fabricated.

## Electronic supplementary material

Supplementry video

Supplementry Information

## References

[1] Lewis NS, Nocera DG (2006). Powering the planet: Chemical challenges in solar energy utilization. Proc. Natl. Acad. Sci..

[2] Yuan S (2016). Integrating 3D Flower-Like Hierarchical Cu2NiSnS4 with Reduced Graphene Oxide as Advanced Anode Materials for Na-Ion Batteries. . ACS Appl. Mater. Interfaces.

[3] Lu X (2013). High energy density asymmetric quasi-solid-state supercapacitor based on porous vanadium nitride nanowire anode. Nano Lett..

[4] Tao J (2015). High-Performance Solid-State Supercapacitors Fabricated by Pencil Drawing and Polypyrrole Depositing on Paper Substrate. Nano-Micro Lett..

[5] Huang, Z., Ma, Y. & Wang, L. Flexible all-solid-state supercapacitors based on polyaniline orderly nanotubes array. *Nanoscale* 193–200, doi:10.1039/c6nr07921k (2016). 10.1039/c6nr07921k 27906390

[6] Simon P, Gogotsi Y (2008). Materials for electrochemical capacitors. Nat. Mater..

[7] Hall PJ (2010). Energy storage in electrochemical capacitors: designing functional materials to improve performance. Energy Environ. Sci..

[8] Wang G, Zhang L, Zhang J (2012). A review of electrode materials for electrochemical supercapacitors. Chem. Soc. Rev..

[9] Feng X (2015). Facile synthesis of shape-controlled graphene–polyaniline composites for high performance supercapacitor electrode materials. New J. Chem..

[10] Zhang LL, Zhou R, Zhao XS (2009). Carbon-based materials as supercapacitor electrodes. J. Mater. Chem..

[11] Gopalakrishnan K, Sultan S, Govindaraj A, Rao CNR (2015). Supercapacitors based on composites of PANI with nanosheets of nitrogen-doped RGO, BC1.5N, MoS2 and WS2. Nano Energy.

[12] Khan Z (2017). Redox-Additive-Enhanced High Capacitance Supercapacitors Based on Co _2_ P _2_ O _7_ Nanosheets. Adv. Mater. Interfaces.

[13] Senthilkumar B (2015). Highly porous graphitic carbon and Ni_2_ P_2_ O_7_ for a high performance aqueous hybrid supercapacitor. J. Mater. Chem. A.

[14] Liao Q, Li N, Jin S, Yang G, Wang C (2015). All-solid-state symmetric supercapacitor based on Co_2_ P_2_ O_7_ nanoparticles on vertically aligned graphene. ACS Nano.

[15] Novoselov KS (2004). Electric Field Effect in Atomically Thin Carbon Films. Science (80-.)..

[16] Chuang C-M, Huang C-W, Teng H, Ting J-M (2010). Effects of Carbon Nanotube Grafting on the Performance of Electric Double Layer Capacitors. Energy & Fuels.

[17] Xu Y (2013). Flexible solid-state supercapacitors based on three-dimensional graphene hydrogel films. ACS Nano.

[18] Minakshi Sundaram M, Biswal A, Mitchell D, Jones R, Fernandez C (2016). Correlation among physical and electrochemical behaviour of nanostructured electrolytic manganese dioxide from leach liquor and synthetic for aqueous asymmetric capacitor. Phys. Chem. Chem. Phys..

[19] Jung N (2013). Synthesis of chemically bonded graphene/carbon nanotube composites and their application in large volumetric capacitance supercapacitors. Adv. Mater..

[20] Chen Q, Zhao Y, Huang X, Chen N, Qu L (2015). Three-dimensional graphitic carbon nitride functionalized graphene-based high-performance supercapacitors. J. Mater. Chem. A.

[21] Lin Z, Taberna PL, Simon P (2015). Graphene-Based Supercapacitors Using Eutectic Ionic Liquid Mixture Electrolyte. Electrochim. Acta.

[22] Mousavi, M. P. S. *et al*. Ionic Liquids as Electrolytes for Electrochemical Double-Layer Capacitors: Structures that Optimize Speci fi c Energy, doi:10.1021/acsami.5b11353 (2016). 10.1021/acsami.5b11353 26771378

[23] Choi BG, Hong J, Hong WH, Hammond PT, Park H (2011). Facilitated ion transport in all-solid-state flexible supercapacitors. ACS Nano.

[24] Xiao X (2012). Fiber-based all-solid-state flexible supercapacitors for self-powered systems. ACS Nano.

[25] Wang, K. *et al*. An All-Solid-State Flexible Micro-supercapacitor on a Chip. **1**, 1068–1072 (2011).

[26] Ke Q, Wang J (2016). Graphene-based Materials for Supercapacitor Electrodes - A Review. J. Mater..

[27] Stoller MD, Park S, Yanwu Z, An J, Ruoff RS (2008). Graphene-Based ultracapacitors. Nano Lett..

[28] Yang X, Zhu J, Qiu L, Li D (2011). Bioinspired effective prevention of restacking in multilayered graphene films: Towards the next generation of high-performance supercapacitors. Adv. Mater..

[29] Huang Y, Liang J, Chen Y (2012). An overview of the applications of graphene-based materials in supercapacitors. Small.

[30] Li F (2009). One-step synthesis of graphene/SnO2 nanocomposites and its application in electrochemical supercapacitors. Nanotechnology.

[31] Yuan L (2012). Flexible solid-state supercapacitors based on carbon nanoparticles/MnO 2 nanorods hybrid structure. ACS Nano.

[32] Wang H (2011). Advanced asymmetrical supercapacitors based on graphene hybrid materials. Nano Res..

[33] Liu C, Yu Z, Neff D, Zhamu A, Jang BZ (2010). Graphene-based supercapacitor with an ultrahigh energy density. Nano Lett..

[34] Qu Q, Yang S, Feng X (2011). 2D sandwich-like sheets of iron oxide grown on graphene as high energy anode material for supercapacitors. Adv. Mater..

[35] Stankovich S (2007). Synthesis of graphene-based nanosheets via chemical reduction of exfoliated graphite oxide. Carbon N. Y..

[36] Pumera M (2011). Graphene-based nanomaterials for energy storage. Energy Environ. Sci..

[37] Ansari SA, Cho MH (2017). Simple and Large Scale Construction of MoS2-g-C3N4 Heterostructures Using Mechanochemistry for High Performance Electrochemical Supercapacitor and Visible Light Photocatalytic Applications. Sci. Rep..

[38] Zhao Y (2015). Well-constructed single-layer molybdenum disulfide nanorose cross-linked by three dimensional-reduced graphene oxide network for superior water splitting and lithium storage property. Sci. Rep..

[39] Cao L (2013). Direct laser-patterned micro-supercapacitors from paintable MoS2 films. Small.

[40] Soon JM, Loh KP (2007). Electrochemical Double-Layer Capacitance of MoS[sub 2] Nanowall Films. Electrochem. Solid-State Lett..

[41] Pumera M, Sofer Z, Ambrosi A (2014). Layered transition metal dichalcogenides for electrochemical energy generation and storage. J. Mater. Chem. A.

[42] Splendiani A (2010). Emerging photoluminescence in monolayer MoS2. Nano Lett..

[43] Yang, X., Niu, H., Jiang, H., Wang, Q. & Qu, F. High energy density all-solid-state asymmetric supercapacitor based on MoS2/graphene nanosheet and MnO2/graphene hybrid electrodes. *J. Mater. Chem. A* 11264–11275, doi:10.1039/C6TA03474H (2016).

[44] Krishnamoorthy K, Pazhamalai P, Veerasubramani GK, Kim SJ (2016). Mechanically delaminated few layered MoS2 nanosheets based high performance wire type solid-state symmetric supercapacitors. J. Power Sources.

[45] Yang C, Dong L, Chen Z, Lu H (2014). High-performance all-solid-state supercapacitor based on the assembly of graphene and manganese(II) phosphate nanosheets. J. Phys. Chem. C.

[46] Acerce M, Voiry D, Chhowalla M (2015). Metallic 1T phase MoS2 nanosheets as supercapacitor electrode materials. Nat. Nanotechnol..

[47] Li N (2012). Ionic liquid assisted hydrothermal synthesis of hollow vesicle-like MoS2 microspheres. Mater. Lett..

[48] Krishnamoorthy K, Veerasubramani GK, Radhakrishnan S, Kim SJ (2014). Supercapacitive properties of hydrothermally synthesized sphere like MoS2 nanostructures. Mater. Res. Bull..

[49] Wu Z, Wang D, Sun A (2009). Surfactant-assisted fabrication of MoS2 nanospheres. J. Mater. Sci..

[50] Li Q, Li M, Chen Z, Li C (2004). Simple solution route to uniform MoS2 particles with randomly stacked layers. Mater. Res. Bull..

[51] Chen X, Li X, Jiang Y, Shi C, Li X (2005). Rational synthesis of α-MnO2 and γ-Mn2O3 nanowires with the electrochemical characterization of α-MnO2 nanowires for supercapacitor. Solid State Commun..

[52] Zallen R, Slade M (1974). Rigid-layer modes in chalcogenide crystals. Phys. Rev. B.

[53] Ramana C (2008). Oxidation and metal-insertion in molybdenite surfaces: evaluation of charge-transfer mechanisms and dynamics. Geochem. Trans..

[54] K N. Kudin *et al*. Raman Spectra of Graphite Oxide and Functionalized Graphene Sheets, doi:10.1021/NL071822Y (2007). 10.1021/nl071822y 18154315

[55] I. Calizo, †, A. A. Balandin, *,†, W. Bao, ‡, F. Miao, ‡ and & Lau‡, C. N. Temperature Dependence of the Raman Spectra of Graphene and Graphene Multilayers, doi:10.1021/NL071033G (2007). 10.1021/nl071033g 17718584

[56] Graf, D. *, † *et al*. Spatially Resolved Raman Spectroscopy of Single- and Few-Layer Graphene, doi:10.1021/NL061702A (2007). 10.1021/nl061702a 17297984

[57] Ellacott MV (1994). Composition of cathode deposits during fullerene production by carbon arc plasma. Carbon.

[58] Shi Y (2013). Self-assembly of hierarchical MoSx/CNT nanocomposites (2 < x < 3): towards high performance anode materials for lithium ion batteries. Sci. Rep..

[59] Kong D (2014). Rational design of MoS2@graphene nanocables: towards high performance electrode materials for lithium ion batteries. Energy Environ. Sci..

[60] Huang K-J (2013). Synthesis of polyaniline/2-dimensional graphene analog MoS2 composites for high-performance supercapacitor. Electrochim. Acta.

[61] Senthilkumar ST (2012). Redox additive aqueous polymer gel electrolyte for an electric double layer capacitor. RSC Adv..

[62] Roshny S (2014). MnO 2 nano/micro hybrids for supercapacitors: ‘Nano’s Envy, Micro’s pride’. RSC Adv..

[63] Ma G (2013). *In situ* intercalative polymerization of pyrrole in graphene analogue of MoS2 as advanced electrode material in supercapacitor. J. Power Sources.

[64] Krishnamoorthy K, Kim S-J (2013). Growth, characterization and electrochemical properties of hierarchical CuO nanostructures for supercapacitor applications. Mater. Res. Bull..

[65] Yang M, Jeong J-M, Huh YS, Choi BG (2015). High-performance supercapacitor based on three-dimensional MoS2/graphene aerogel composites. Compos. Sci. Technol..

[66] Li, N. *et al*. Compact graphene/MoS_2_ composite films for highly flexible and stretchable all-solid-state supercapacitors. *J. Mater. Chem. A* 3267–3273, doi:10.1039/C6TA10165H (2017).

[67] Yang H (2011). Influences of graphene oxide support on the electrochemical performances of graphene oxide-MnO2 nanocomposites. Nanoscale Res. Lett..

[68] Electrochemistry, S. S. Effect of graphitic structure on electrochemical ion intercalation into positive and negative electrodes Effect of graphitic structure on electrochemical ion intercalation into positive and negative electrodes, doi:10.1007/s10008-014-2527-7 (2014).

[69] Fang M (2009). Covalent polymer functionalization of graphene nanosheets and mechanical properties of composites. J. Mater. Chem..

[70] Zhang, H., Bhat, V. V., Gallego, N. C. & Contescu, C. I. Thermal Treatment E ff ects on Charge Storage Performance of Graphene-Based Materials for Supercapacitors (2012). 10.1021/am300593k 22680779

[71] Muniraj VKA, Kamaja CK, Shelke MV (2016). RuO2??nH2O Nanoparticles Anchored on Carbon Nano-onions: An Efficient Electrode for Solid State Flexible Electrochemical Supercapacitor. ACS Sustain. Chem. Eng..

[72] Karbhal, I. *et al*. Facile Green Synthesis of BCN Nanosheets as High-Performance Electrode Material for Electrochemical Energy Storage. *Chem. - A Eur. J*. 7134–7140, doi:10.1002/chem.201505225 (2016). 10.1002/chem.201505225 27072914

[73] Anothumakkool B (2016). High-Performance Flexible Solid-State Supercapacitor with an Extended Nanoregime Interface through *in Situ* Polymer Electrolyte Generation. ACS Appl. Mater. Interfaces.

[74] Soni R, Anothumakkool B, Kurungot S (2016). 1D Alignment of PEDOT in a Buckypaper for High-Performance Solid Supercapacitors. ChemElectroChem.

[75] Rathod D (2009). Design of an ‘all solid-state’ supercapacitor based on phosphoric acid doped polybenzimidazole (PBI) electrolyte. J. Appl. Electrochem..

[76] Hastak RS, Sivaraman P, Potphode DD, Shashidhara K, Samui AB (2012). All solid supercapacitor based on activated carbon and poly [2,5-benzimidazole] for high temperature application. Electrochim. Acta.

[77] Sivaraman P (2010). All solid supercapacitor based on polyaniline and crosslinked sulfonated poly[ether ether ketone]. Electrochim. Acta.

[78] Tamilarasan P, Ramaprabhu S (2013). Graphene based all-solid-state supercapacitors with ionic liquid incorporated polyacrylonitrile electrolyte. Energy.

[79] Liu Y (2015). High-Performance Flexible All-Solid-State Supercapacitor from Large Free-Standing Graphene-PEDOT/PSS Films. Sci. Rep..

