# Supplementary material for: Three-dimensional Graphene with MoS2 Nanohybrid as Potential Energy Storage/Transfer Device
Source: Sci Rep. 2017 Aug 25;7:9458. doi: 10.1038/s41598-017-09266-2 (PMC5573343; doi:10.1038/s41598-017-09266-2)
Supplement: Supplementary file 2 — Supplementry Information [file 41598_2017_9266_MOESM2_ESM.pdf]

Supplementary Information

Three-Dimensional Graphene with MoS_2 Nanohybrid as Potential Energy Storage/Transfer Device

*Kulvinder Singh, Sushil Kumar, Kushagra Agarwal, Khushboo Soni, Venkata Ramana Gedela and Kaushik Ghosh** ***(kaushik@inst.ac.in)***

Institute of Nano Science and Technology, Sec. 64, Mohali Punjab

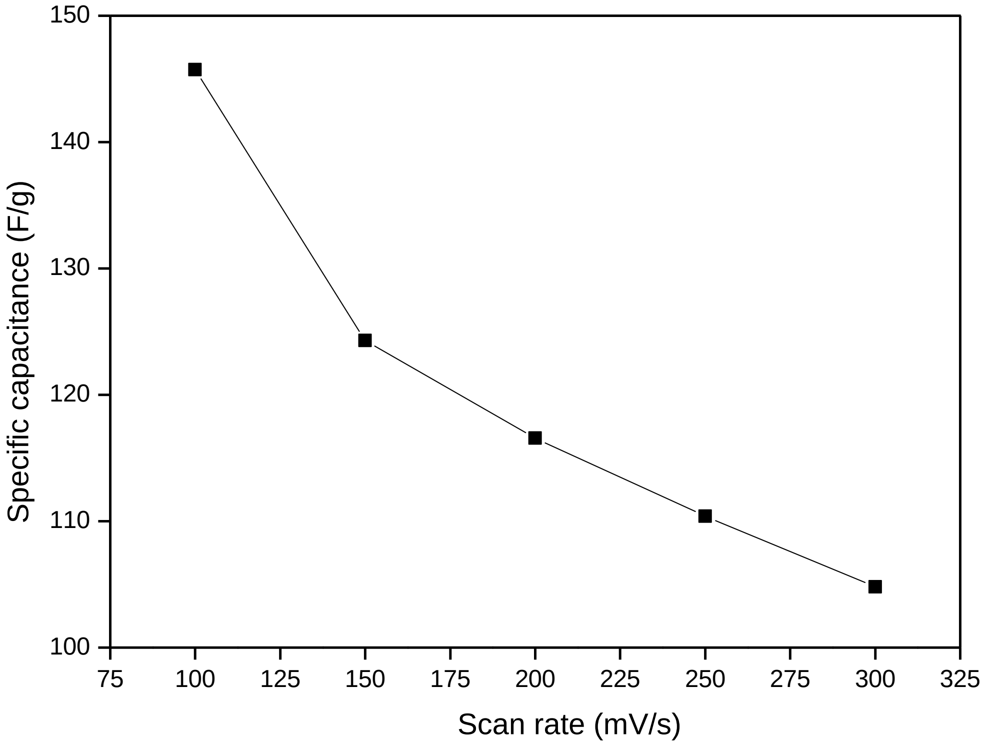

Figure S1 Effect of scan rate on specific capacitance of MoS_2 nanoflowers

Supplementary Information

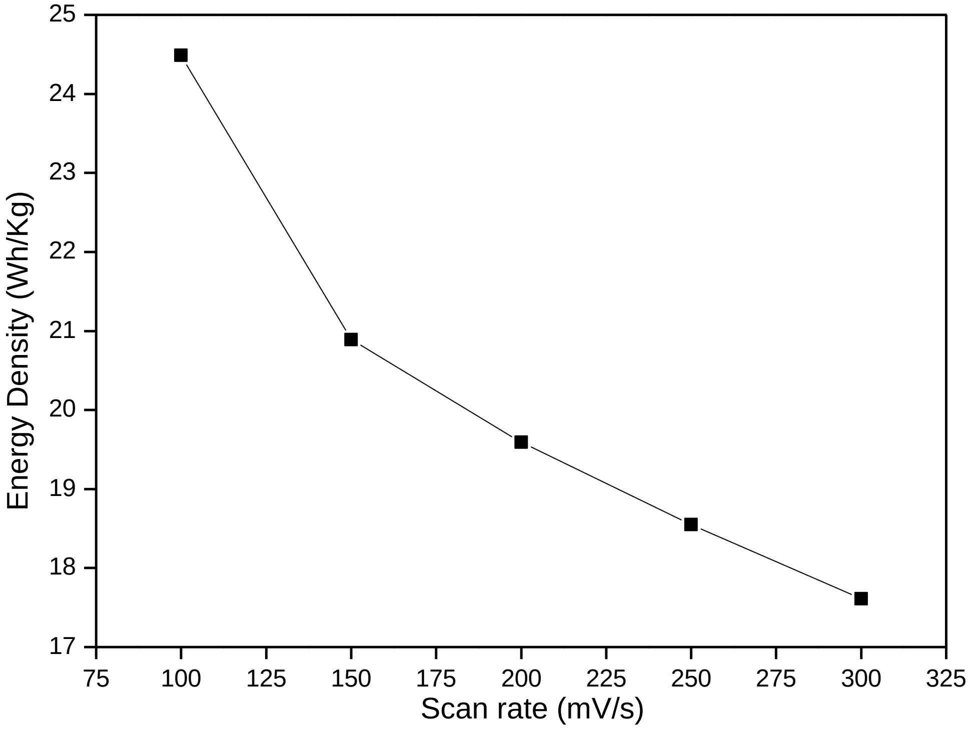

Figure S2 Effect of scan rate on energy density of MoS_2 nanoflowers

Supplementary Information

Table 1 Effect of scan rate on specific capacitance and Energy density of MoS_2 nanoflowers

Scan rate (mV/s)	100	150	200	250	300
Specific capacitance (F/g)	145.73	124.31	116.58	110.39	104.79
Energy density(Wh/Kg)	24.49	20.89	19.59	18.55	17.61

Table 2 Effect of Current density on specific capacitance, Energy density and power density of MoS_2 nanoflowers

Current Density (A/g)	1	2	3	4
Specific capacitance (F./g)	169.37	95.66	62.52	46.17
Energy density(Wh/Kg)	28.43	16.07	10.5	7.75
Power density (W/Kg)	10.18	8.36	30.88	36.90

Table 3 Table Effect of scan rate on specific capacitance of solid state device

Scan rate (mV/s)	50	100	150	200	250	300	350	400	450	500
Specific capacitance (F/g)	55.09	43.69	39.38	33.68	31.09	28.85	27.12	25.57	24.53	23.55

Supplementary Information

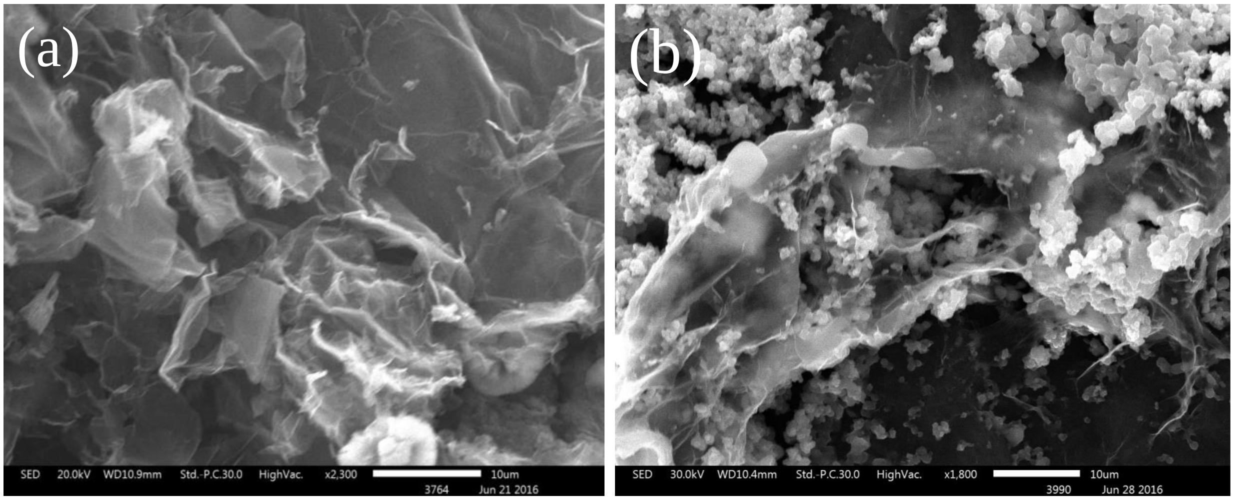

Figure S3: (a) The image depicts the presence of 3D graphene architecture on graphite sheet, (b) the corresponding MoS_2 nanoflowers dispersion on the 3D graphene structure.

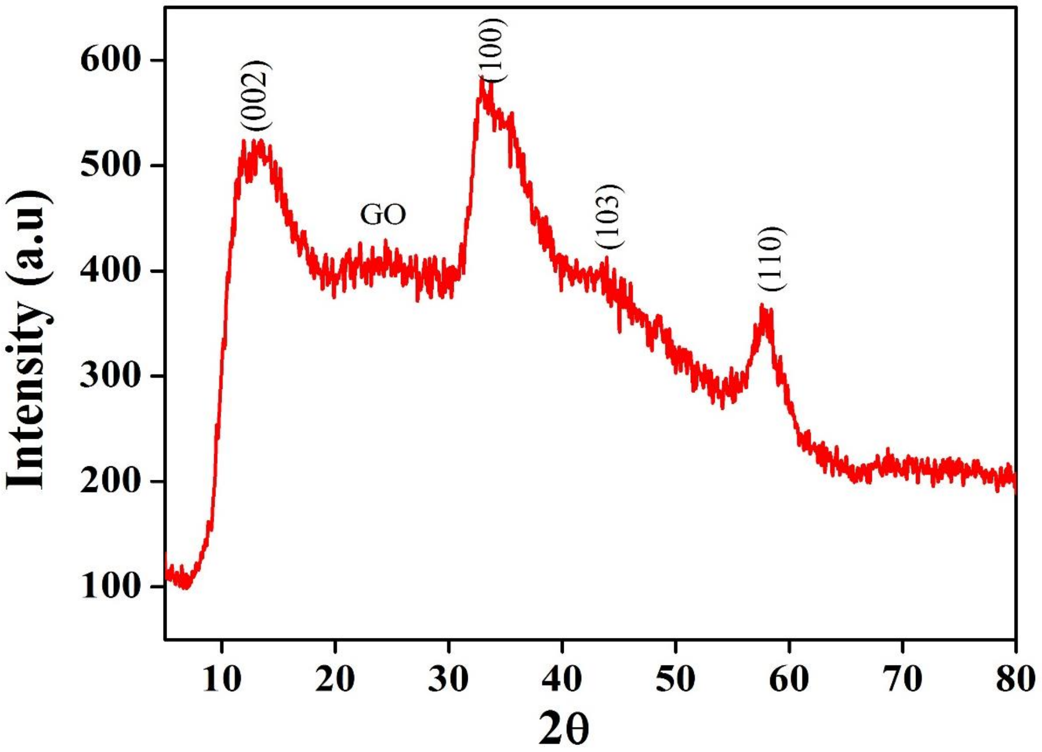

Figure S4: The characteristic diffraction pattern of as-prepared 3D graphene-MoS_2 hybrid material, where the presence of graphitic network and MoS2 nanoflakes are simultaneously present which is in agreement with the recent finding[56].

Supplementary Information

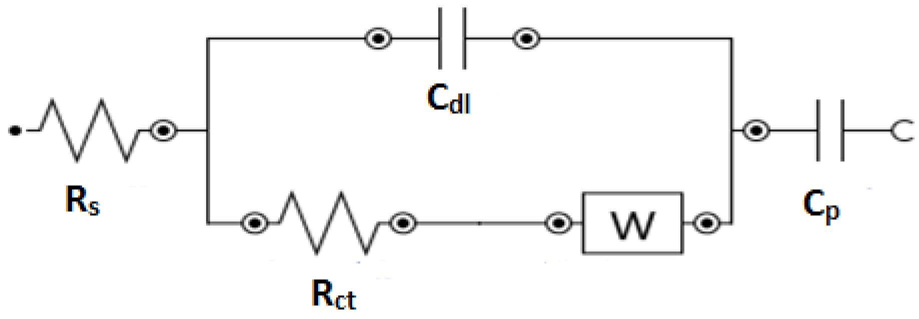

Figure S5: Simulated circuit for fitting the EIS data.

Here Rs is the internal resistance, R_{ct} charge transfer resistance, C_{dl} double-layer capacitor, C_p limit capacitance, W warburg impedance. By fitting the data in this simulated circuit, we calculate the Rs value, which is comes out to be 18.66 ohm.
